# Algorithm for biological second messenger analysis with dynamic regions of interest

**DOI:** 10.1371/journal.pone.0284394

**Published:** 2023-05-11

**Authors:** Jennifer M. Knighten, Takreem Aziz, Donald J. Pleshinger, Naga Annamdevula, Thomas C. Rich, Mark S. Taylor, Joel F. Andrews, Christian T. Macarilla, C. Michael Francis

**Affiliations:** 1 Department of Physiology and Cell Biology, University of South Alabama College of Medicine, Mobile, Alabama, United States of America; 2 Department of Pharmacology, University of South Alabama College of Medicine, Mobile, Alabama, United States of America; 3 Center for Lung Biology, University of South Alabama College of Medicine, Mobile, Alabama, United States of America; 4 Bioimaging Core Facility, University of South Alabama College of Medicine, Mobile, Alabama, United States of America; Cinvestav-IPN, MEXICO

## Abstract

Physiological function is regulated through cellular communication that is facilitated by multiple signaling molecules such as second messengers. Analysis of signal dynamics obtained from cell and tissue imaging is difficult because of intricate spatially and temporally distinct signals. Signal analysis tools based on static region of interest analysis may under- or overestimate signals in relation to region of interest size and location. Therefore, we developed an algorithm for biological signal detection and analysis based on dynamic regions of interest, where time-dependent polygonal regions of interest are automatically assigned to the changing perimeter of detected and segmented signals. This approach allows signal profiles to be rigorously and precisely tracked over time, eliminating the signal distortion observed with static methods. Integration of our approach with state-of-the-art image processing and particle tracking pipelines enabled the isolation of dynamic cellular signaling events and characterization of biological signaling patterns with distinct combinations of parameters including amplitude, duration, and spatial spread. Our algorithm was validated using synthetically generated datasets and compared with other available methods. Application of the algorithm to volumetric time-lapse hyperspectral images of cyclic adenosine monophosphate measurements in rat microvascular endothelial cells revealed distinct signal heterogeneity with respect to cell depth, confirming the utility of our approach for analysis of 5-dimensional data. In human tibial arteries, our approach allowed the identification of distinct calcium signal patterns associated with atherosclerosis. Our algorithm for automated detection and analysis of second messenger signals enables the decoding of signaling patterns in diverse tissues and identification of pathologic cellular responses.

## Introduction

Cells use soluble second messengers including calcium ions (Ca^2+^), cyclic adenosine monophosphate (cAMP), cyclic guanosine monophosphate, and nitric oxide to transduce external stimuli into physiologic responses [1–3]. Signal specificity is achieved by compartmentalization of these messengers spatially in relation to effectors and the time course or frequency of second messenger elevation [2, 4–6]. Techniques to measure dynamic intracellular second messenger concentrations have improved, with spatial resolution approaching the limits of light microscopy, and high-speed sampling enables measurement of nanosecond signals [7–10]. However, substantiation of signal specificity through direct observation of spatially and temporally regulated signals is limited because of inadequate analytical methods to accommodate microscopy data; for example there is no current analytical standard for time-resolved volumetric imaging data [11–15].

Manually decoding the multiple intracellular signaling events in cultured cell monolayers and intact tissue using available analysis techniques is time consuming and subjective, and the resultant datasets often are large and multidimensional and may include 3-dimensional time-lapse measurements with spectral information from multiple fluorophores [10]. Measurements rely on the analysis of the static region of interest (ROI), and signals are identified visually and delimited manually by polygonal ROIs to measure the mean signal intensity over time within the region [13–23]. Available methods of second messenger signal analysis typically are limited in the degree of automation, but automation has become necessary because of the large amount of signaling data generated with current microscopy systems. In addition, available methods are not generalizable and rely on static ROI analysis. There is a need for a fully automated method for second messenger signal analysis that satisfies the typical use cases of investigators including both hyperspectral and volumetric time lapse imaging data that accommodates the breadth of signaling behavior exhibited by biological systems.

We previously developed a standardized, algorithmic method for signal detection with static ROIs that enables the rigorous and rapid automated detection of signal regions [18]. This method revealed the functional tuning of second messenger signals to stimuli in cell fields via recruitment of actively signaling cells and modulation of signal intensity, duration, spatial spread, and frequency proportional to distinct functional responses [6, 24]. This technique also revealed distinct tissue-wide characteristic signatures of endothelial cell calcium signaling in multiple vascular beds [25–27]. These calcium signatures are amplified in proportion to functional responses within the vasculature and altered distinctly in various diseases including carotid injury [28] and pulmonary arterial hypertension [26]. However, ROI analysis inherently is limited because of common approaches of constrained ROI shapes to preserve comparable signal measurements and the static characteristics of ROIs. Static regions underestimate signals that are much smaller than the region perimeter and overestimate signals that exceed the region.

We developed a new method, known as the S8 algorithm, for automated signal detection and characterization for second messenger analysis that uses dynamic region tracking and facilitates the precise detection of cellular signals with varying contours over time, without the limitations of predefined or estimated ROIs. We designed and validated this automated algorithm that is independent of input data structure, such as 3-dimensional, time-lapse, or spectral information data, and uses dynamic ROIs that track the changing location of detected signals over time rather than within a static ROI perimeter. The purpose of the present study was to describe the S8 algorithm in mathematical notation, validate the algorithm using synthetic ground truth datasets of multiple varieties, and test important use cases for the algorithm with multidimensional and human datasets in which stimulus-response coupling was needed with signal specificity in physiological and pathological scenarios.

## Methods

### Software implementation

The S8 algorithm was implemented in the Python Language (Python Software Foundation. Python Language Reference, version 3.8.5. Available at http://www.python.org), and dependent on the following packages: Numpy (version 1.19.2), Trackpy (version 0.4.2), Matplotlib (version 3.3.2), Plotly (version 4.14.3), Psutil (version 5.7.2), PIMS (version 0.5), Scikit-image (version 0.17.2), Scipy (version 1.5.3), Tifffile (version 2020.10.1), and Tqdm (version 4.50.2). Installation and usage instruction can be found at: https://github.com/franccm/s8.

### Ground truth data

Ground truth validation data were generated manually and with a programming language (GNU Octave, v5.1.0) as 2 distinct datasets: (1) arbitrary dynamic polygonal data in the absence of noise and (2) Gaussian signal pulses embedded in various levels of random noise. To construct the former noiseless dataset, 15 image sequences were manually created (ImageJ, National Institutes of Health, Bethesda, MD) that contained 57 dynamic signal events at multiple locations within the image stacks. These dynamic signal events, defined by contiguous successive polygonal regions of various intensity, included scenarios of concentric growth and decay, signal propagation defined by continuous unidirectional movement in the xy plane, periodicity, and signal convergence (signals merging) and divergence (signal locale splitting).

To examine algorithm dependence on signal-to-noise ratio, image stacks were generated (GNU Octave) containing 181 frames with embedded normally distributed background noise (mean ± standard deviation, 1 ± 1 arbitrary units). Gaussian signal pulses were embedded in each image stack using a method (ode23, MathWorks, Natick, MA) to solve for signal intensity over time, given a Gaussian rate equation with a peak at frame no. 90 in a 30-pixel^2^ area located in the center of the image stack. The peak height of the signal pulse was varied from an intensity of 0 to 10 arbitrary units in increments of 0.5 arbitrary units, corresponding to signal-to-noise-ratios from 0 to 10. Maximal spatial spread at peak signal intensity was computed as ground truth for each signal-to-noise ratio value greater than 0.

### Volumetric time-lapse hyperspectral cAMP measurements

Rat microvascular endothelial cells were cultured as previously described [29], separated, and placed in Dulbecco Modified Eagle Medium. The cells were transferred to round coverslips at 37°C and grown to 70% to 80% confluency. At 48 h before imaging, the cells were transfected with an H188 fluorescence resonance energy transfer (FRET) sensor [30]. At 10 min before imaging, the cells were labeled with a fluorescent nuclear label (DRAQ5, ThermoFisher, Waltham, MA). The coverslips with adherent cells were placed in a cell chamber (Attofluor, ThermoFisher) and bathed in 800 μL buffer containing 145 mM NaCl, 4 mM KCl, 20 mM HEPES, 10 mM d-glucose, 1 mM MgCl2, and 1 mM CaCl2, at pH 7.3. Additional buffer (200 μL) containing a sufficient concentration of isoproterenol was added to bring the concentration of isoproterenol to 1.0 μM in the cell chamber.

Spectral imaging microscopy was performed as previously described [31]. Transfected cells were imaged using a confocal microscope (A1R, Nikon, Melville, NY) with a water immersion objective (magnification ×60; Plan Apo VC 60x DIC N2 WI NA-1.2, Nikon) and 32-channel spectral detector. Axial image stacks (z-stacks) were acquired every 30 seconds for 20 minutes. The donor (H188: Turquoise-cAMP Binding domain from Epac-Venus) was excited at 405 nm (laser intensity, 8%, equivalent to 1.82 μW at the sample stage), and the nuclear label (DRAQ5) was excited at 561 nm (intensity, 10%, equivalent to 19.02 μW at the sample stage). Spectral emission was measured from 414 to 724 nm in 10 nm increments. The cells were treated with isoproterenol at 1 min after acquisition of baseline emission.

Spectral image analysis was performed as previously described [31]. Images were unmixed using a custom script (MATLAB, MathWorks) and smoothed using Gaussian filtering. FRET efficiency was calculated and mapped to cAMP concentration using the Hill equation (Hill coefficient, 1). Correlation analysis of signal peak time vs z-position was performed using R (The R Foundation for Statistical Computing, version 3.4.1 “Single Candle”, Vienna, Austria) correlation test with the Pearson method.

### Calcium measurements in excised arteries

Mesenteric arteries were isolated and dissected from freshly euthanized mice. All procedures were approved by the South Alabama institutional animal care and use committee. Human neurovascular bundle segments were removed from the limbs of patients who had undergone amputation surgery for advanced cardiovascular disease or severe limb trauma. All tissues were acquired after complete processing and evaluation by the University of South Alabama College of Medicine Department of Pathology and consent was waived by the ethics committee. Patient information was deidentified, and sex, age, and the presence or absence of diagnosed peripheral arterial disease and diabetes were recorded. Tissues were stored in HEPES-buffered physiological saline solution (HPSS: sodium chloride, 134 mM; potassium chloride, 6 mM; magnesium chloride, 1 mM; calcium chloride, 2 mM; HEPES, 10 mM; glucose, 10 mM; pH, 7.45) at 4°C and transported to the laboratory for study. Branches from the posterior tibial artery (3–5 artery segments; diameter, 0.5–5 mm) were dissected and used in the experiments within 8 to 18 hours after amputation. All procedures were approved by the University of South Alabama Institutional Biosafety Committee.

Artery segments (mesenteric or tibial) were opened longitudinally with microscissors and mounted flat (intima facing upward) onto rectangular silicone inserts (SYLGARD, Dow, Midland, MI) with tungsten micropins. The tissues were incubated with Ca^2+^ indicator loading solution containing calcium dye (Cal-520 AM, Abcam, Cambridge, UK) (10 μM) at room temperature for 40 minutes in the dark. After washing and equilibration (30 min), inserts were inverted and placed in a glass-bottom chamber containing HPSS (tissue, 100 μm from glass). The chamber was mounted on a spinning disk confocal inverted microscope (Andor Revolution, Andor, Belfast, UK), and Ca^2+^-dependent fluorescence was measured at 20 or 8 frames/s, mesenteric or tibial arteries, respectively, at 25°C using image analysis software (Andor iQ, Andor) (magnification ×20; 1024 × 1024 pixels) (wavelength: excitation, 488 nm; emission, 510 nm). Differences between control and diseased groups log area (μm^2^) distributions were quantified via Student’s t-test.

## Results

### Description of the method

We defined the input image stack U(c, t, z, y, x) as an array of intensity values for channel c, time t, height z, width y, and length x:

$$\text{U}\left(\text{c},\text{t},\text{z},\text{y},\text{x}\right)$$ (1)

A smoothed version U_smooth_ of the data represented by U was generated by applying the Savitzky-Golay smoothing function [32] to the image stack for each c, z, y, and x pixel element over time t, with Savitzky-Golay_t_ being a function of polynomial order (set to 3) and window size specified as the optimal window for Nyquist sampling:

$${\text{U}}_{\text{smooth}}={\text{Savitzky-Golay}}_{\text{t}\left(\text{order},\text{window}\right)\text{U}}$$ (2)

Background subtraction was performed:

$${\text{U}}_{\text{filtered}}={\text{U}}_{\text{smooth}}–{\text{U}}_{\text{smooth}}\left({\text{min}}_{\text{t}}\right)$$ (3)

where min_t_ was the minimum intensity of every pixel over time. The smoothed and background subtracted image stack was used to create a binary mask by blurring and performing adaptive thresholding before computing the Hadamard product with the filtered image stack:

$$\text{V}=\text{Gaussian}\;\text{Blur}\left(\text{sigma}\right){\text{U}}_{\text{filtered}}\;$$ (4)

$${\text{V}}_{\text{binary}}=\text{Adaptive}\;\text{Threshold}\;\text{V}$$ (5)

where V was the filtered image (U_filtered_) blurred by an xy Gaussian Blur of magnitude sigma, and V_binary_ was a binary image sequence of V after adaptive thresholding was applied using the Otsu method [33]. Here, either Yen [34] or Triangle [35] adaptive thresholding approaches may also be employed for more or less stringency, respectively. U_mask_ was a masked image created by multiplying the filtered image U_filtered_ by V_binary_:

$${\text{U}}_{\text{mask}}={\text{U}}_{\text{filtered}}\;\times {\text{V}}_{\text{binary}}$$ (6)

The accumulated images of U_mask_ and V_binary_ were rendered by summing each image over time:

$${\text{V}}_{\text{sum}}=\Sigma {\text{V}}_{\text{binary}}$$ (7)

$${\text{U}}_{\text{sum}}=\Sigma {\text{U}}_{\text{mask}}$$ (8)

where Σ was the element-wise sum of all values of the V_binary_ and U_mask_ images. Contiguous particles in the summed arrays were identified using the Python package (Scikit-image; https://scikit-image.org/), defining discrete 4-dimensional regions of interest (x, y, z, t). These regions were defined as sites because they corresponded to the locale encompassed by overlapping second messenger signals. Dynamic ROIs were defined by temporally contiguous particle cross-sections along the time axis. Volumetric and slicewise descriptors such as mean intensity, area, duration, starting slice, and bounding box were calculated, and 4-dimensional particles were associated with their corresponding time-dependent slicewise measurements using a custom, in-house Python function (3Tracker). A directory of sites and corresponding signal properties were rendered as a compressed Python dictionary file, and signal properties were visualized using interactive graphics (Plotly, Dash, Montreal, Canada).

### Validation

**Fig 1:**
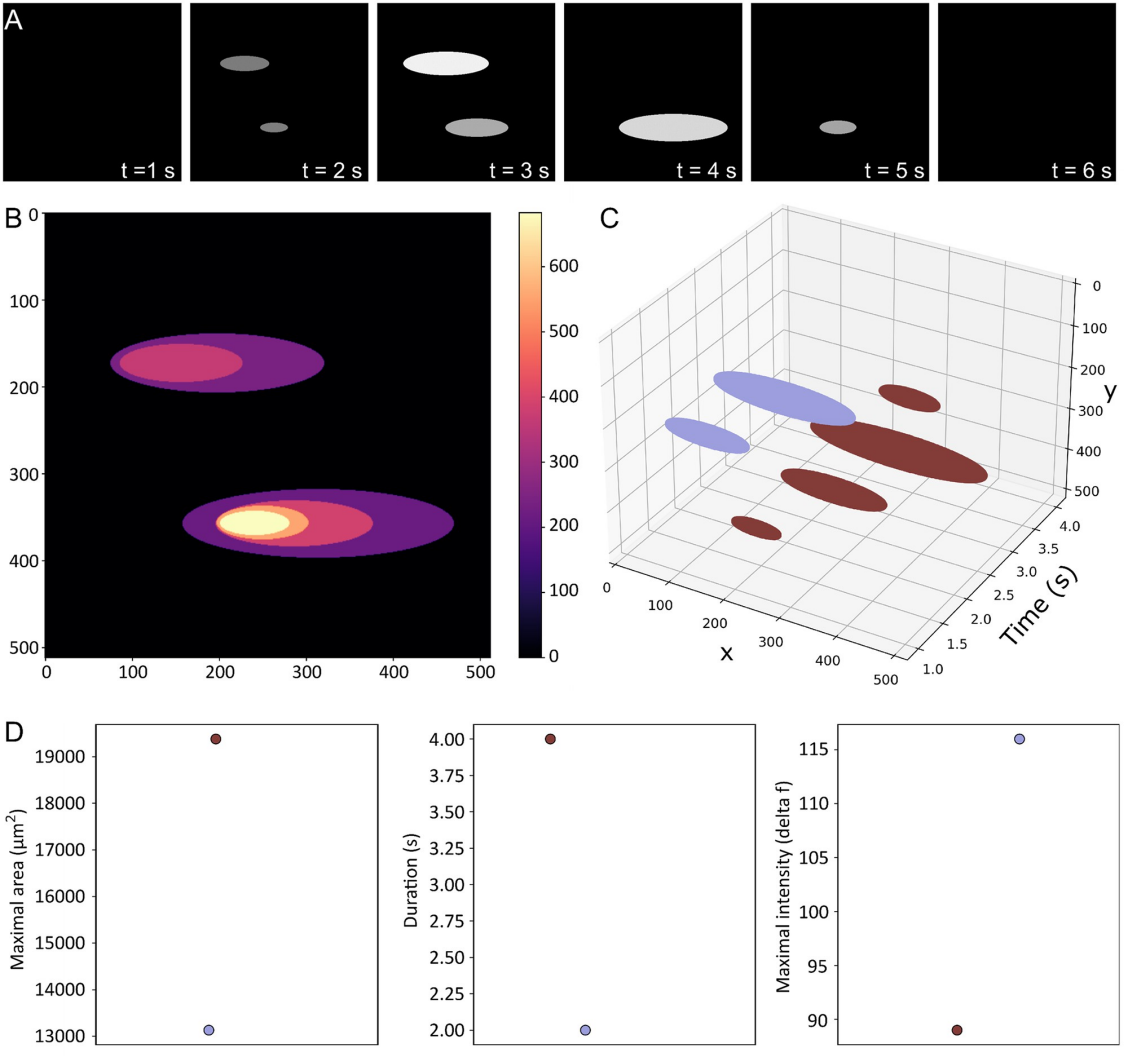
Particle tracking validation of the S8 algorithm. **(A)** A binary movie was generated (ImageJ) in which circles with varying areas in the x-y plane represented a diffuse signal changing over time from left to right (time between images, 1 s). **(B)** The pseudosignal area and location over time were detected correctly using S8, and the sum of particles from all frames. **(C)** The pseduosignal time course was displayed in a heat map that detected dynamic regions over time. **(D)** With time represented on the z-axis, S8 produces graphical output showing area and location of the signal over time.

In order to validate our method for second messenger signal analysis, we formulated 2 distinct datasets for (1) validation of signal segmentation and (2) evaluation of signal identification as a function of signal-to-noise ratio. For the first dataset, image processing software (ImageJ) was used to create 3-dimensional image stacks in which a binary signal was embedded in a static background. This dataset enabled the evaluation of S8 algorithm for identification and gross level classification performance in the absence of noise. Both 8-bit and 16-bit 3-dimensional image stacks were created, which tested the ability of the S8 algorithm to detect and classify static signals, spatially and temporally dynamic signals, waves, oscillations, and convergent and divergent signals. The segmentation dataset resulted in an ellipsoid locus of white pixels, encoded in a black background, that changed over time (Fig 1). The input image stack shows the location of particles comprising two contiguous signals over time (Fig 1A), and the algorithmically labeled signal was shown as a 3-dimensional time-lapse sequence (Fig 1B). For all image stacks, the algorithm correctly labeled the ground truth embedded in the dataset.

**Fig 2:**
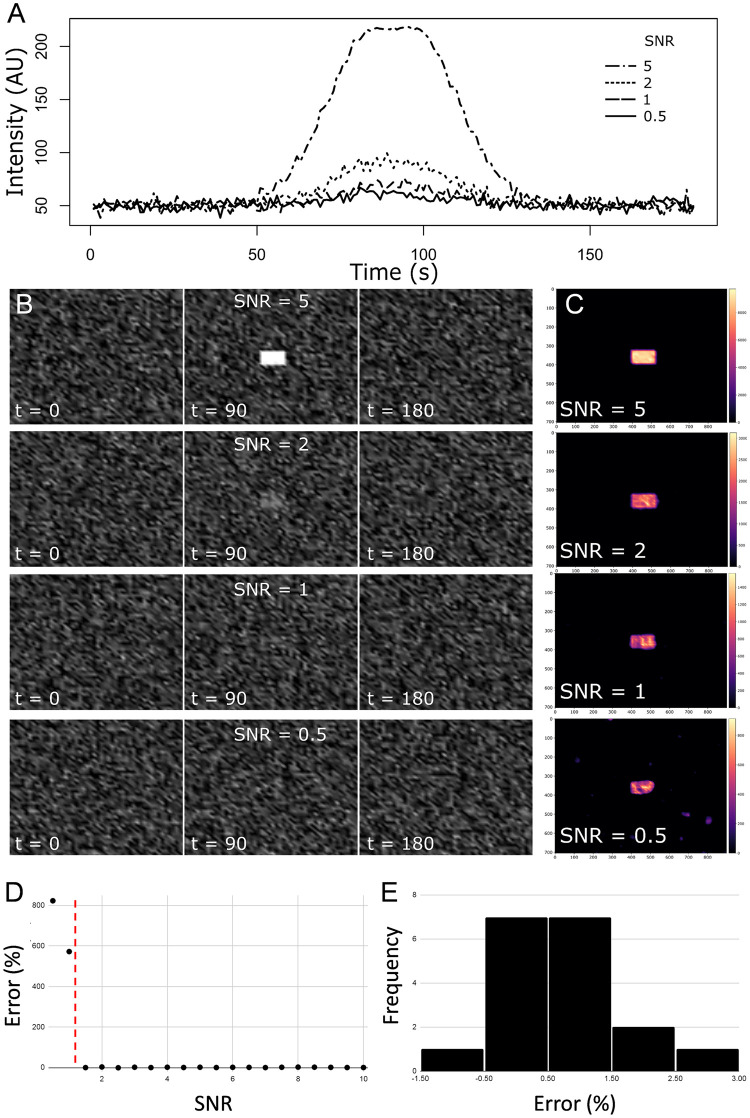
Evaluation of automated signal detection in noisy data with S8 algorithm. **(A)** Intensity vs time graphs show the amplitude of Gaussian signal pulses embedded in background noise. SNR, signal-to-noise ratio. **(B)** Selected images from time (t) = 0, 90, and 180 seconds showing the position (center image) of the signal pulse at peak intensity for signal-to-noise ratio of 5, 2, 1, and 0.5. **(C)** S8 output of the filtered and summed signal showing the location of the ground truth signal pulse, detected in **B**. **(D)** Percent error of ground truth was low for signal-to-noise ratios > 1 (indicated by the vertical dashed line). **(E)** Percent error was normally distributed (mean ± SD, 0.71 ± 1.01).

Evaluation of signal detection as a function of signal-to-noise ratio was performed using a dataset of 3-dimensional image stacks in which a Gaussian signal pulse was encoded in random uniform background noise (MATLAB), adapting a technique described previously (Fig 2A) [18]. The signal-to-noise ratio (SNR) was varied from 0.5 to 10 to determine the sensitivity of the technique to noise. Fig 2B, middle panel shows the peak intensity of 4 representative signals with SNR of 5, 2, 1, and 0.5, respectively, and the signal pulse ground truth area was identified correctly in every image stack (Fig 2C), although the frequency of false positives in the image stacks increased with SNR greater than 0.5. Fig 2D shows percent error of ground truth as a function of SNR (0.5–10), where percent error approaches 0 for SNR greater than 1 (vertical red line). A histogram of percent error (Fig 2E) shows that for SNR greater than 1, error values are normally distributed around 0.71 ± 1.01 (mean ± standard deviation).

**Fig 3:**
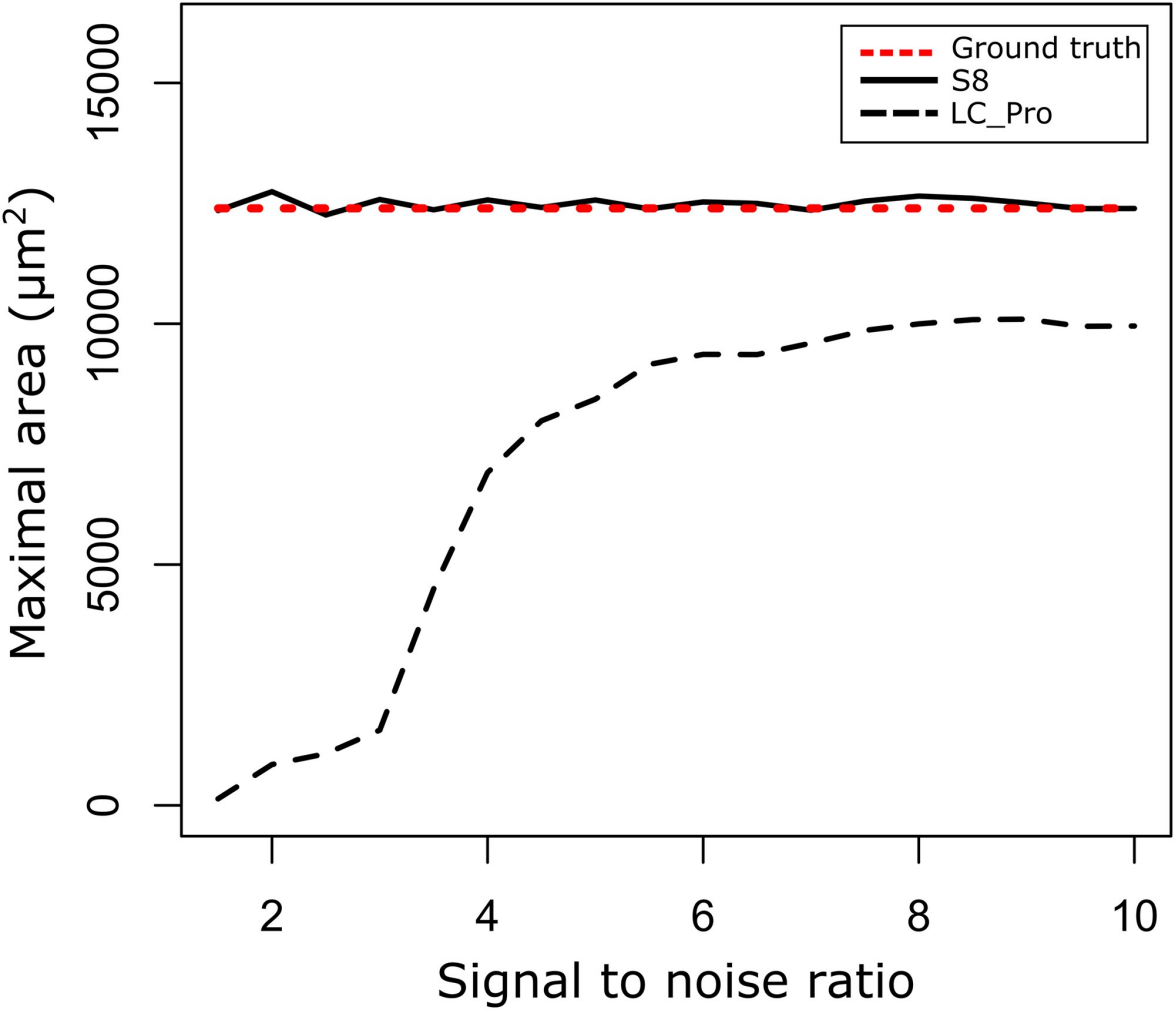
Comparison between S8 and LC_Pro signal detection algorithms of the maximal area of signals embedded in random noise with signal to noise ratios (SNR) from 2–10. The S8 algorithm (solid black line) consistently and accurately detected the maximal area of the ground truth signal (red dashed line) within the noisy test dataset for SNR of 2–10. LC_Pro (black dashed line) drastically underestimates the maximal area at SNR of 2–4, while systematically underestimating the signal within SNR of 7–10 compared to S8.

Next, we compared S8 to our previous algorithm for static ROI detection, LC_Pro, by evaluating the detected ground truth maximal spatial area in noisy data as a function of signal-to-noise ratio (Fig 3). We found that for signal-to-noise ratios above 2, S8 accurately detected the ground truth, while LC_Pro systematically underestimated the ground truth area, especially for low signal-to-noise ratios (<4).

### Description of feature set

Literature searches for automated and semiautomated second messenger analysis solutions showed 40 methods described previously [15, 19, 21, 22]. Table 1 lists unique features of S8 in comparison to methods that are publicly available. Second messenger signals are commonly frequency-modulated and investigators measure this phenomenon by measuring signal oscillation frequency, Fourier analysis, and determination of signal initiation sites which may be associated with pacemaker cell activity. Thus, output data from S8 includes a python dictionary indexed by detected signal with corresponding information on signal dynamic ROI composition, duration, frequency, and origination sites compiled into a graphical report. Importantly, because S8 utilized time-dependent (dynamic) ROIs, normalized (f/f0) signal intensity measurements are not possible. Instead, maximal change in intensity (delta f) is a reported metric of signal amplitude.

**Table 1:** Features of S8 listed with other automated and semiautomated signal analytics software by degree of automation, support for multidimensional images, and characterization of signal phenomena.

Feature	S8	LC_Pro	CALIMA	SIMA	SparkMaster	CellProfiler
Fully automated	Yes	No	No	No	NA	Yes
Dynamic ROI	Yes	No	No	No	NA	No
2-d sequences	Yes	Yes	Yes	Yes	NA	Yes
3-d sequences	Yes	No	No	No	NA	No
Waves	Yes	No	No	No	NA	No
Oscillation frequency	Yes	Yes	No	Yes	NA	Yes
Normalized intensity	No	Yes	Yes	Yes	Yes	Yes

***Abbreviations*:** 2-d, 2-dimensional; 3-d, 3-dimensional; NA, not applicable, ROI, region of interest.

### Endothelial calcium measurements in opened mouse mesenteric arteries

**Fig 4:**
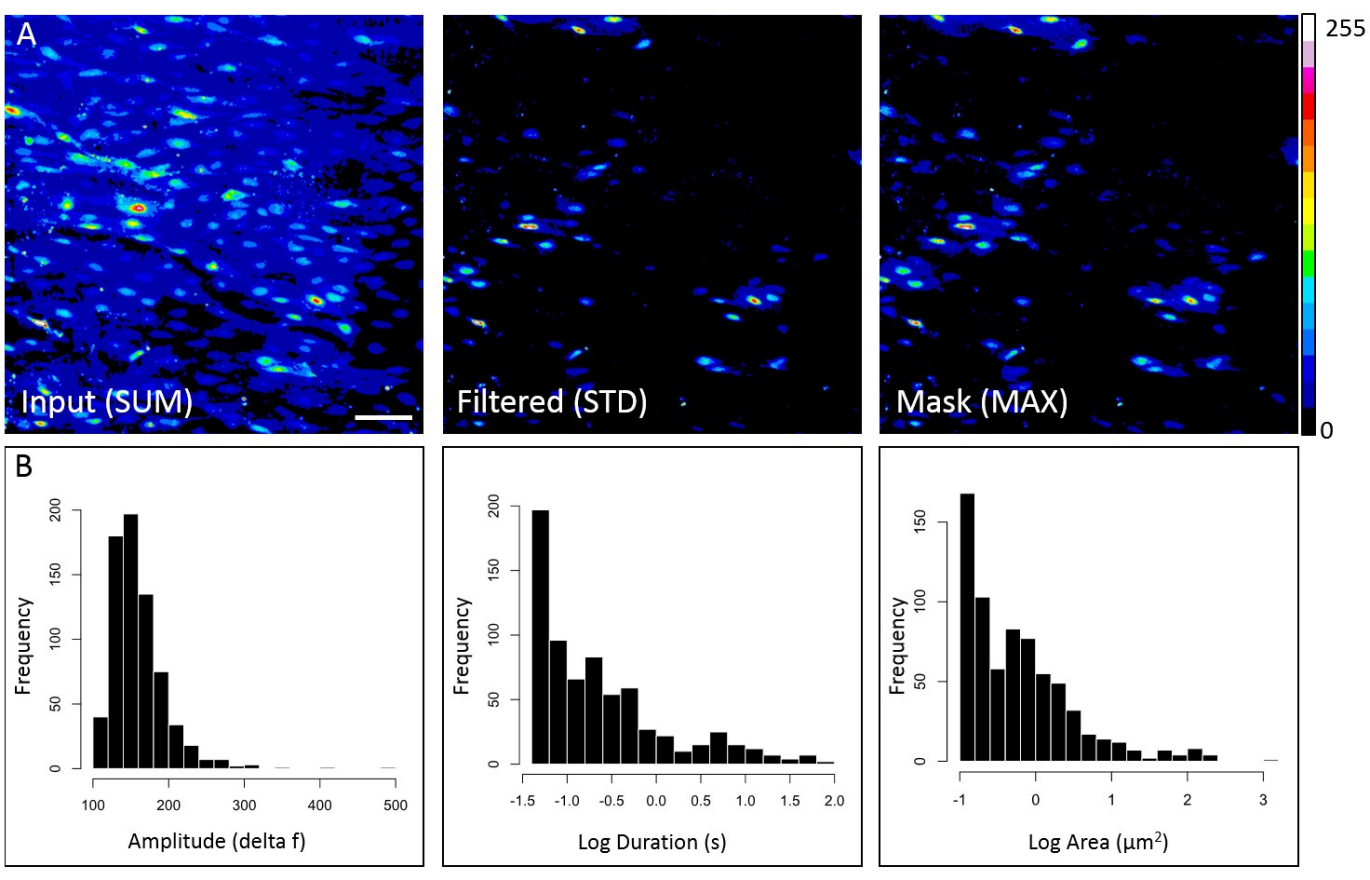
S8 analysis of calcium activity in mouse mesenteric artery endothelium. **(A)** Pseudo-colored images represent the time-dependent sum (SUM) of the raw input image sequence (left), the standard deviation (STD) of the filtered image sequence highlighting cellular calcium activity, and the maximum projection (MAX) of calcium events detected by S8 in the output mask. Scale bar = 50 μm. **(B)** Histograms showing the distributions of maximal amplitude (delta f), log duration (s), and log maximal area (μm^2^) for calcium events detected in mouse mesenteric endothelial cells.

The *ex vivo* application of the S8 algorithm was showcased by analyzing endothelial calcium activity in mouse mesenteric arteries prepared using the open artery method and loaded with Cal-520 (Fig 4). The sum of the input image sequence shows a confluent monolayer of vascular endothelial cells (Fig 4A, left). After S8 executed the filtering module comprised of a per-pixel Savitzky-Golay smoothing and background subtraction, the standard deviation of the filtered image sequence revealed areas of variable intensity over time representing regions of calcium activity (Fig 4A, center). The maximum intensity projection of the S8-generated mask (Fig 4A, right), shows regions of automatically detected endothelial calcium signaling events. Histograms show the distributions of maximal delta amplitude (left), log duration (center), and log maximal area (right) for events detected in mouse mesenteric vascular endothelial cells.

### Volumetric time-lapse hyperspectral cAMP measurements

**Fig 5:**
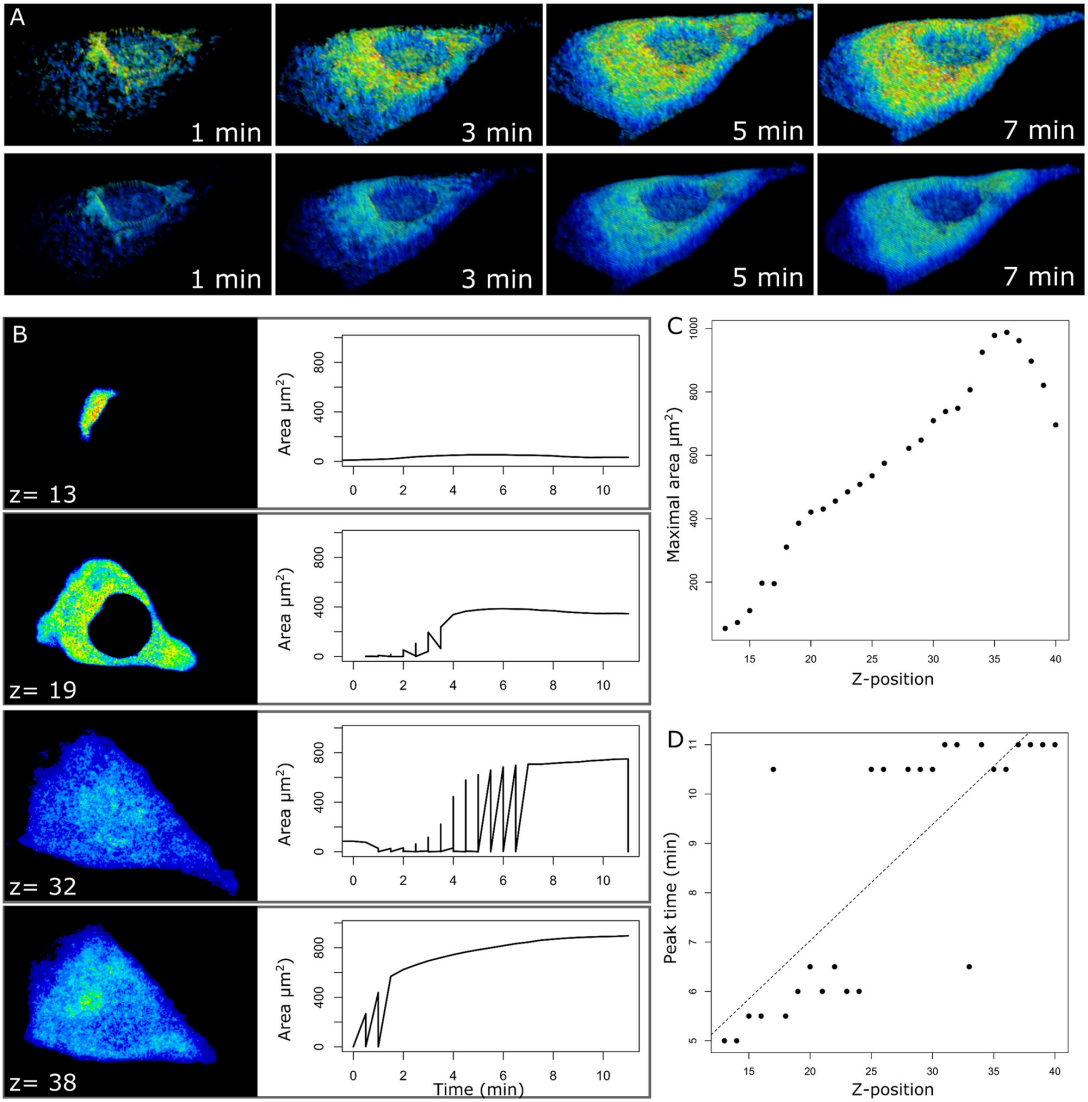
Analysis of hyperspectral cyclic adenosine monophosphate (cAMP) data with S8 algorithm. **(A)** Unmixed hyperspectral images of cAMP-dependent fluorescence resonance energy transfer (FRET) in a rat microvascular endothelial cell show the time course of an isoproterenol-induced cAMP signal before (top row) and after (bottom row) S8 filtering and background subtraction. **(B)** Cross-sectional analysis of the time-resolved volume scan shows signal heterogeneity with respect to the apical-basolateral cell axis (z = 13, 19, 32, 38). Area vs time plots of associated dynamic regions of interest reveal distinct signals. **(C)** Quantification of maximal spatial area shows signal amplification and a delay to peak toward the basal cell surface (z position = 40). **(D)** Quantification of signal peak time (min) shows positive correlation with z-position, indicating a delay to peak signal as a function of cell depth (Pearson test: *P* = 1.41 × 10^−6^).

To demonstrate a high-dimensional analysis use case, intracellular cAMP was measured by successive volumetric hyperspectral scans in cultured rat microvascular endothelial cells transfected with a FRET probe (Fig 5). After spectral unmixing and conversion from relative FRET intensity to cAMP-dependent fluorescence, time-resolved volume scans were analyzed automatically with the S8 algorithm. A single smooth muscle cell was selected for evaluation of isoproterenol-induced signal responses as a function of depth along the apical-basal cell axis. Noise and static signal were subtracted from raw images by S8 using the Savitzky-Golay algorithm and background subtraction (Fig 5A). Cross-sectional signals and associated plots of area vs time for detected dynamic ROIs revealed signal heterogeneity as a function of cell depth after isoproterenol was added to the apical cell surface (Fig 5B). Maximal signal area was amplified as a function of cell depth, indicating intracellular expansion of the isoproterenol-induced signal toward the basal cell surface (Fig 5C). Signal time to peak area increased as a function of cell depth, indicating longer response times with distance from the stimulus (*P* = 1.41 × 10^−6^), possibly due to sedimentation of the drug in the extracellular solution (Fig 5D). These results confirm the application of automated analysis to 5-dimensional image sequences (length, width, height, time, channel).

### Characterization of atherosclerotic signals in human arteries

**Fig 6:**
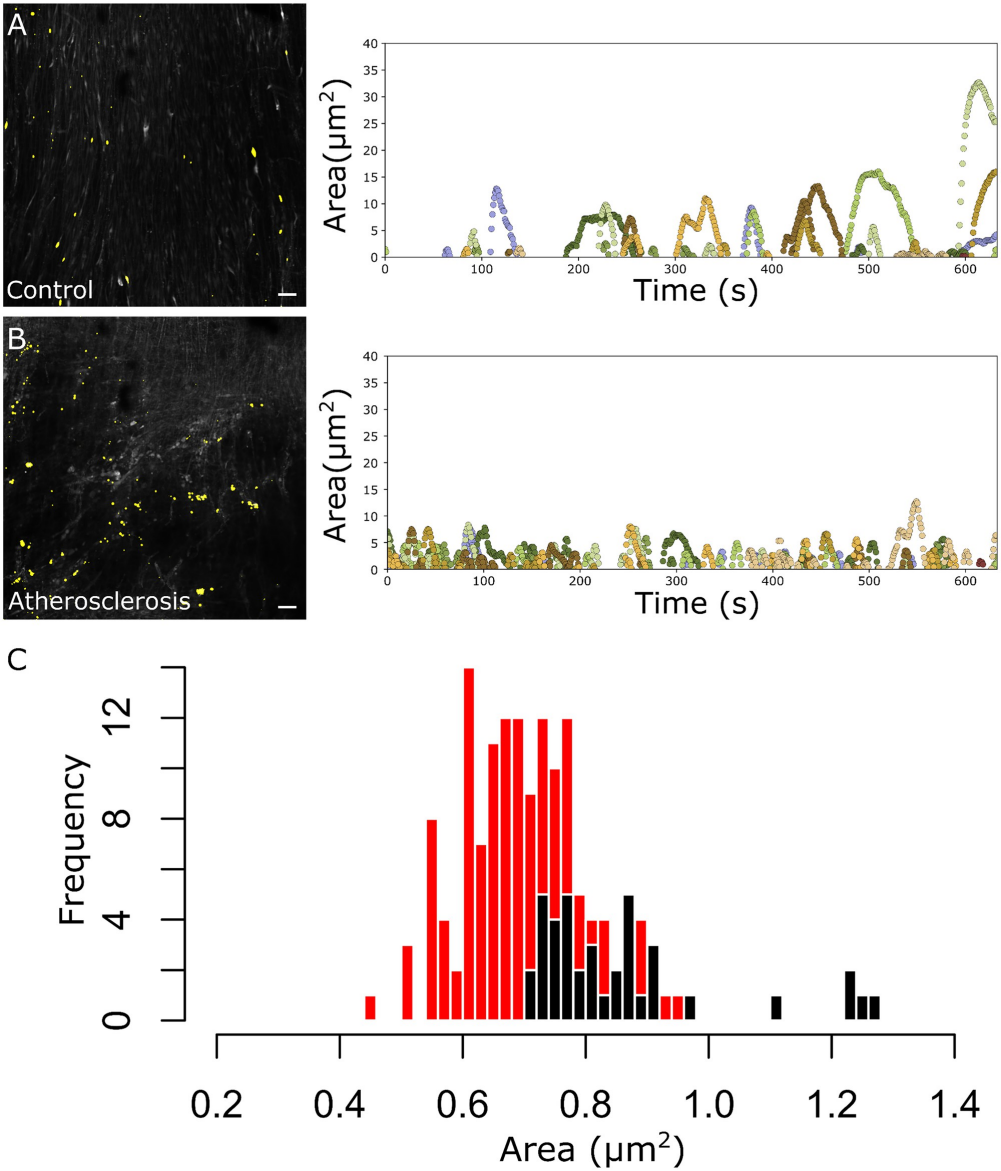
Signal patterning in atherosclerosis with S8 algorithm. **(A and B)** Human tibial arteries were dissected and mounted en face. Endothelial calcium signals were measured with Cal-520 and processed with S8. Time-lapse images of 5 min of unstimulated activity are shown (left panels) with automatically detected dynamic regions of interest highlighted in yellow. Tracings of each region of interest location (right panel) show dynamic signal area over time, color-coded by region. Scale bar = 30 μm. **(A)** Control patient. **(B)** Patient with atherosclerosis. **(C)** Histograms of log maximal signal area for control (black) and atherosclerosis patients (red) reveal distinct distributions associated with disease (*t* test: *P* = 9.99 × 10^−8^).

To demonstrate the application of the S8 algorithm to the characterization of pathological signal patterns, S8 was applied to image sequences of calcium signals from isolated human tibial arteries prepared with the open artery technique (Fig 6A). S8 analysis revealed distinct signal associated with detected dynamic ROIs in atherosclerotic vs control samples (Fig 6B). Histograms of aggregate signaling patterns defined by maximal signal area revealed distinct, normal log scale distributions that defined a pathologic calcium signal phenotype in atherosclerotic samples (*P* = 9.99 × 10^−8^) (Fig 6C). These results confirmed the utility of the S8 algorithm in defining signal signatures in diseased tissue.

## Discussion

The present method for biological signal analysis relied on dynamic ROIs rather than the static ROI methods described previously. While the distribution of amplitude, duration, and spatial spread was consistent with previous *ex vivo* studies, the S8 algorithm consistently outperformed LC_Pro by accurately detecting signaling events. The use of dynamic ROIs enabled computation of the spatial area encompassed by second messenger signals as it changed over time. This is important because signal specificity is achieved by the relationship between the signal and effect or locale. This method may enable the quantification of the dynamic signal in relation to effectors via optical microscopic methods such as immunofluorescence microscopy. Importantly, although our approach utilizes background subtraction to extract transient cell signals, it does not preclude the identification of longer, global changes in second messenger concentration within image stacks.

The information that may be derived from S8 analysis is not specific to a single category or classification of events. The method holistically encompasses a broad range of second messenger signal patterns. The importance of second messenger signals and the organization of disparate signals into characteristic signal profiles or signatures may enable the characterization of fundamental physiological and pathological processes in individual cells. In contrast with other methods of automated or semi-automate second messenger analysis, the S8 algorithm uses dynamic ROIs that enable the tracking of spatial spread over time. The S8 algorithm also enables 4- and 5-dimensional image stack analyses, in contrast with other methods that are confined to line scan images [36, 37] or 2-dimensional time-lapse image data structures [17, 19, 21, 22].

The S8 software also revealed second messenger signals that may not otherwise be visible because of low signal-to-noise ratio. The Savitzky-Golay noise filtering algorithm at Nyquist frequency enabled high-fidelity temporal filtering and minimization of image artifact [38]. In addition, the higher dimensional capabilities of S8 enabled the measurement of z-axis spatial heterogeneity of second messenger signals within single cells, as shown in our analysis of rat microvascular endothelial cells after isoproterenol treatment. However, it should be noted that there are issues with correlation of signals from time resolved volumetric data, as the sequential nature of z-plane scanning and time-dependent measurements mean that such data are not a true representation of spatially-resolved temporal signals. Technological advances in high speed confocal microscopy in the future may minimize these concerns.

A limitation of this study was that the mean fluorescence intensity in a static ROI was not applicable because we implemented dynamic ROIs, and this may necessitate revised interpretations of comparable measurements of signal amplitude between multiple static ROIs. The concept of signal amplitude, as defined by fold change of the mean intensity of an ROI from baseline over time, was no longer valid with the current method, and a revised definition of signal amplitude may be needed. The change in amplitude over the course of an event may obscure the fact that a single small punctate bright signal may appear functionally equivalent to a larger, broad, dim event. As we cannot define the signal baseline based on a static ROI with the S8 algorithm, fluorescence intensity amplitude cannot be scaled in the conventions used by other available methods.

Another phenomenon related to cell signaling is the apparent convergence and divergence of cell signaling sites. For example, multiple cells may generate signals which coincide at specific spatial local or single sites may generate propagating waves in multiple directions. Importantly, the usage of dynamic ROIs with S8 allows the automatic tracking and delineation of both convergent and divergent signals, as signals that coincide but originate or terminate in non-overlapping regions may be identified by the shape of the “volumetric” ROI. Such signals are flagged as convergent or divergent by the software depending on their temporal orientation and are then associated with their “daughter” or “parent” events, respectively.

The present results justify the future development of a dashboard interface to construct biobanks of the second messenger signals analyzed by the S8 algorithm. Raw output data may be stored and evaluated to address issues of replicability and provide opportunities for meta-analysis. In addition, recent machine learning approaches have been advanced to overcome the artifacts associated with imaging data to provide rapid and automatic signal segmentation [39]. These approaches may be integrated into the S8 image processing pipeline. Furthermore, emergent behaviors may occur in tissues, defined as coordinated behaviors that are linked temporally and spatially. The vascular endothelium may function as a syncytium of cells and integrate disparate intracellular signals into smooth, continuous, and coordinated responses. Temporal coherence may occur between disparate second messenger signals, and the endothelium and other tissues may be modeled as functional systems analogous to neural networks [40]. The S8 analysis method may enable standardized and generalized quantitation of such phenomena.

In summary, the S8 algorithm is a dynamic ROI tracking software that integrates available image processing pipelines for noise filtering and image digitization. This algorithm may enable improved elucidation of the mechanisms of second messenger signal specificity by filtering image noise, addressing image artifacts, amplifying endogenous second messenger signals without introducing false positive signals, and generalization for diverse intracellular second messenger signal events. The S8 algorithm is available as open source software (https://drive.google.com/file/d/1n-M0eGhGH4pniRzIPEjNH1RMh4ETIUDy/view?usp=sharing) and may be integrated into other available analytics methods.
